# Heritability of Sleep and Its Disorders in Childhood and Adolescence

**DOI:** 10.1007/s40675-021-00216-z

**Published:** 2021-11-22

**Authors:** Katie J. S. Lewis, Alice M. Gregory

**Affiliations:** 1 Division of Psychological Medicine & Clinical Neurosciences, MRC Centre for Neuropsychiatric Genetics & Genomics, Cardiff University School of Medicine, Hadyn Ellis Building, Maindy Road, CF24 4HQ Cardiff, UK; 2 Department of Psychology, Goldsmiths, University of London, London, UK

**Keywords:** Adolescence, Childhood, Circadian rhythms, Heritability, Sleep

## Abstract

**Purpose of Review:**

This review summarizes recent literature on the heritability of sleep and sleep disorders in childhood and adolescence. We also identify gaps in the literature and priorities for future research.

**Recent Findings:**

Findings indicate that age, measurement method, reporter, and timing of sleep measurements can influence heritability estimates. Recent genome-wide association studies (GWAS) have identified differences in the heritability of sleep problems when ancestral differences are considered, but sample sizes are small compared to adult GWAS. Most studies focus on sleep variables in the full range rather than on disorder. Studies using objective measures of sleep typically comprised small samples.

**Summary:**

Current evidence demonstrates a wide range of heritability estimates across sleep phenotypes in childhood and adolescence, but research in larger samples, particularly using objective sleep measures and GWAS, is needed. Further understanding of environmental mechanisms and the interaction between genes and environment is key for future research.

## Introduction

In recent years, there have been rapid advances in our understanding of the heritability of sleep phenotypes in humans, but research has primarily focused on adults. Significant developmental changes in sleep physiology and behaviors occur across childhood and adolescence [1–3], and this might have implications for estimates of heritability of sleep across the lifespan. In this review, we provide a brief overview of childhood and adolescent sleep, followed by a summary of methods used to assess sleep and heritability. We then focus on literature published within the last 5 years on the heritability of sleep and its disorders in childhood and adolescence. Finally, we highlight areas of interest for future research.

## Sleep and Sleep Disorders in Childhood and Adolescence

Numerous lines of evidence suggest that sleep is important for children and adolescents, including for their physical and mental health, cognitive processing, and educational attainment [1, 4]. The dominant theory posits that sleep timing and duration are regulated by two processes [5]: *Process S*, a homeostatic process in which “sleep pressure” increases during waking hours and dissipates during sleep, and *Process C*, circadian (approximately 24-h) peaks and troughs in arousal. Process S and Process C interact to promote sleep onset when sleep pressure is highest and circadian timing for arousal is low. Research on the neurobiological underpinnings of the two-process model is ongoing, but current evidence suggests that Process S is linked to the accumulation of certain molecules including adenosine in the brain [6] and that Process C is primarily controlled by the suprachiasmatic nuclei, a collection of neurons in the anterior hypothalamus [7].

After sleep onset, in adults, the neurophysiology of sleep can be divided into two main states: non-rapid eye movement (NREM) sleep and rapid eye movement (REM) sleep. NREM sleep typically follows sleep onset, and is divided into 3 stages (N1, N2, and N3) [8]. N1 and N2 are lighter stages of sleep, whereas N3 (also known as deep sleep or slow-wave sleep) is characterized by low-frequency, high-amplitude brain activity. K-complexes and sleep spindles (short bursts of 12–14 Hz activity [9]) typically occur in N2. REM sleep typically occurs after progression through stages N1–N3 (and then back to N2). It is characterized by rapid eye movements, muscle paralysis, and high-frequency, low-amplitude brain activity resembling wakefulness, and is the stage of sleep during which vivid dreaming is most likely [10]. Progressing through one cycle of sleep typically takes 50–60 min in infants [11], which then lengthens to 90–110 min in adults and is repeated 4–6 times during the night [12].

Comprehensive accounts of developmental changes in sleep timing and neurophysiology have been reviewed elsewhere [3, 13, 14]. When individuals transition from childhood to adolescence, sleep timing becomes more delayed [15]. This has been linked to puberty onset and is thought to be partly due to sleep pressure accumulating more slowly throughout the day (however, the dissipation of sleep pressure appears to remain the same as in childhood) [2, 14, 16]. Sleep architecture also changes, with reductions in REM sleep from infancy to childhood, and reductions in slow-wave sleep and slow-wave activity after the onset of puberty [13]. There are also other psychological and socio-cultural influences on sleep during adolescence, such as greater autonomy over bedtimes and changes in social behavior [16].

### Sleep Disorders

The International Classification of Sleep Disorders (ICSD-3) [17] groups sleep disorders under the seven categories (insomnia, sleep-related breathing disorders, central disorders of hypersomnolence, circadian rhythm sleep–wake disorders, sleep-related movement disorders, and other sleep disorders). These are summarized in Table 1.

**Table 1:** Summary of International Classification of Sleep Disorders-3rd Edition (ICSD-3) sleep disorder categories

Category	Example disorders
Insomnia	*Behavioral insomnia of childhood:*
	• Difficulty initiating or maintaining sleep despite adequate opportunity
	• Poor sleep quality with daytime impairment
	• Symptoms present at least three times per week for at least 3 months
	Additional specifiers:
	• *Sleep onset association* — child associates their ability to fall asleep with inappropriate environmental stimuli, such as watching television or falling asleep in the car. This means that when that stimulus is absent, the child experiences difficulties falling asleep
	• *Limit setting insomnia* — child refuses to go to bed or employs tactics to stall bedtime
Central disorders of hypersomnolence	Excessive daytime sleepiness (either prolonged sleep duration or sleepiness) that is not caused by another sleep disorder or circadian rhythm misalignment. Disorders in this section include:
	• *Narcolepsy* — characterized by unintentional sleep bouts, short REM sleep onset latency, and sometimes cataplexy (temporary loss of muscle tone)
	• *Idiopathic hypersomnia* — hypersomnia that cannot be explained by another condition
	• *Kleine-Levin syndrome* — a rare disorder primarily affecting adolescent males [75] characterized by recurrent episodes of excessive sleep, accompanied by “binge eating” and hypersexuality
Sleep-related breathing disorders	Abnormal respiration during sleep. Sleep apnea occurs when breathing temporarily stops during sleep, resulting in reduced blood oxygen saturation, sleep fragmentation, and daytime sleepiness. Three types of sleep apnea are:
	• *Obstructive sleep apnea (OSA)* — caused by obstruction of the upper airways. *Symptoms of pediatric OSA* — snoring, labored/obstructed breathing, or daytime consequences such as sleepiness or hyperactivity to be present
	• *Central sleep apnea (CSA)* — caused by a reduced drive to breath
	• *Mixed* — symptoms of both OSA and CSA
Circadian rhythm sleep–wake disorders (CRSD)	Occur when there is a misalignment between the circadian sleep–wake cycle and the external environment (e.g., socially defined timing of school, work, or social activities)
	• The misalignment can occur because sleep onset is later (delayed sleep–wake phase disorder) or earlier (advanced sleep–wake phase disorder) than is desired, although other forms of CRSD exist
	• The misalignment must be accompanied by insomnia or excessive daytime sleepiness, distress, or impairment, and must last for at least 3 months (with the exception of jet lag disorder) [17]
Sleep-related movement disorders	Movements that prevent or disturb sleep. These disorders include:
	• *Restless legs syndrome (RLS)* — an urge to move the legs that is sometimes accompanied by an uncomfortable sensation. This is partially or totally relieved by movement and occurs primarily in the evening or night
	• *Periodic limb movement disorder (PMLD)* — limb movements that occur during sleep (more than 5 times per hour in children, more than 15 times per hour in adults) which are accompanied by sleep disturbance or other functional impairment
Parasomnias	Physical events or experiences occurring during sleep or in the transition to/from sleep. They can be grouped into the following categories:
	• *NREM parasomnias* — also known as disorders of arousal from NREM, these commonly occur during slow-wave sleep (N3) and include sleepwalking, confusional arousals, sleep terrors, and sleep-related eating disorder
	• *REM parasomnias —* include REM sleep behavior disorder, sleep paralysis, and nightmare disorder
	• *Other parasomnias —* occur during either NREM or REM sleep, or during wakefulness soon after sleep. They include exploding head syndrome and sleep enuresis (bed wetting)
	NREM parasomnias such as sleepwalking are more common in children than adults and are often considered a normal part of development [76, 77]. However, the persistence of these parasomnias past a particular age is considered problematic. For example, sleep enuresis is only considered atypical when it persists after 5 years of age

Note. There is an additional section in the ICSD-3 which focuses on sleep disorders that cannot be classified elsewhere

## Measuring Sleep

Subjective measures of sleep include sleep diaries and questionnaires, which can be collected in both parents/caregivers and children. Actigraphy is often used as an objective measure of sleep and requires participants to wear a device (typically on their wrist or leg) which detects movement. Algorithms are then used to estimate periods of sleep and wakefulness. These algorithms are validated using the “gold-standard” objective measure of sleep, polysomnography (PSG), in which information from electroencephalography (EEG), electrooculography, and electromyography are combined to determine the presence of the sleep stages described above [1]. Videography and other objective measures are also available [18].

## Heritability

Children differ greatly in terms of their sleep, both for genetic and environmental reasons. Heritability is defined as “the proportion of phenotypic differences among individuals that can be attributed to genetic differences in a particular population” [19]. A heritability estimate is specific to the population from which it was derived, and therefore varies depending on the characteristics of that sample. Heritability for psychological and behavioral traits is rarely (if ever) estimated to be 100% heritable [20]. This is partly because measurement error is larger for these traits compared to physical traits such as height, but also reflects the influence of the environment.

Common misconceptions about heritability outlined by Visscher et al. [21] are summarized below:

1. *Heritability is the proportion of a phenotype that is passed on to the next generation*: Actually, genes are passed on, not phenotypes. This is the most common misconception and is common even amongst those who are educated in genetics [22].
2. *High heritability implies genetic determination*: Instead, a high heritability indicates that the phenotype of an individual is a good predictor of genotype. However, due to environmental influences, phenotype is not determined by genotype alone.
3. *Low heritability implies no additive genetic variance*: Instead, low heritability means that of all observed variation, a small proportion is caused by variation in genotypes.
4. *Heritability is informative about the nature of between-group differences*: This is incorrect, actually heritability should not be used to predict changes in a population over time or differences between groups because heritability is defined for a particular population in a particular environment and therefore cannot be applied to other populations which may have different environments.
5. *A large heritability implies genes of a large effect*: This is not the case, and it is more common for high heritability estimates to be due to a polygenic effect (multiple genes), which individually explain a low proportion of variance.

### Family and Adoption Studies

Family studies sample individuals in a population who have a disorder and assess the risk for the disorder in their relatives. A traditional family design cannot be used to disaggregate genetic from environmental effects because family members usually share both genetic and environmental influences [23, 24]. We will therefore not review studies using this method. In contrast, adoption studies can disaggregate the effects of genetics and environment because they can compare the degree of similarity between people who share different amounts of genetic and environmental similarity (e.g., the adoptees and their adoptive and biological parents).

### Twin Studies

Twin studies can be used to estimate heritability by comparing monozygotic (identical) twins, who share 100% of their genes, to dizygotic (non-identical) twins, who share, on average, 50% of their segregating genes. Heritability is estimated by comparing the degree of similarity for a trait (e.g., sleep duration) in monozygotic twin pairs compared to dizygotic twin pairs [25]. Four sources of variance can be estimated: *Additive* genetic influences (“A”); *Dominant* (nonadditive) genetic influences (“D”), *Common* (shared) environmental influences (“C”) which make family members more alike; and *Non-shared Environmental* influences (“E”), which make family members less alike (and includes measurement error) [19]. Using a standard twin design, it is not possible to estimate D and C in the same model; therefore, ACE and ADE models are examined separately.

### Genome-Wide Association Studies (GWAS)

GWAS use a case–control method in samples from populations to detect associations between genetic markers (called single-nucleotide polymorphisms, SNPs) and a disease or trait of interest across the genome [26]. From this, SNP heritability (h^2^_SNP_) can be estimated, which is the proportion of variance in liability associated with common SNPs genome-wide. There are several methods to estimate SNP heritability from GWAS data, each with different assumptions [27]. In contrast to twin studies, in which heritability represents the proportion of variance due to common and rare genetic variants, SNP heritability represents the proportion of variance due to common genetic variants. This is one reason why SNP heritability is usually lower than heritability estimated from twin studies [27].

## Heritability of Sleep and Sleep Disorders in Childhood and Adolescence

### Twin Studies

In recent years, amongst the different techniques used to inform about heritability, twin studies have provided the most information about sleep and sleep disorders in children and adolescence. These studies focus on a plethora of sleep phenotypes.

#### Sleep Duration

Kocevska et al. [28••] meta-analyzed data from 19 twin studies (*N* = 43,328, age 6 months to 88 years) assessing sleep duration using self-report, parent reports, actigraphy, and polysomnography. Heritability of sleep duration varied by age, ranging from 17% in infancy to 69% in adolescence (46% across all ages). Heritability estimates also varied according to the method of measuring sleep and by reporter, with lower estimates for parent reports (8%) vs. self-reports (38%) or sleep diary (52%). These discrepancies were also found by Breitenstein et al. [29••] who found that the heritability of sleep duration in 8-year-old children (*N* = 608) measured using actigraphy was 81% compared to 21% for parent reports.

Inderkum and Tarokh [30] assessed sleep duration for 6 months in 51 adolescents (11–14 years) using actigraphy and self-reports. Heritability estimates for sleep duration measured using actigraphy were 15% on school days but 68% on free days (weekends, public holidays, and vacations) and 45% on holidays (public holidays and vacations). However, self-reported sleep duration was 19% on school days and 2% on free days. The more similar estimates between sleep duration on school days as compared to free days could be explained by the fact that correlations between subjective and objective measures were higher on school days (0.53) compared to free days (0.25). The authors hypothesized that sleep duration may be more accurately recalled for school days than free days because bedtimes and risetimes are likely to be influenced by school start times.

#### Sleep Quality

Kocesvska et al. [28••] meta-analyzed data from 13 twin studies of sleep quality (*N* = 43,328, age 16–82 years). Sleep quality was assessed using self-report (primarily using the Pittsburgh Sleep Quality Index [31]). Two of the studies were conducted in adolescents (age 16–18) with heritability estimates ranging from 33 to 41%. The heritability estimate across all ages was 44%, which did not differ significantly by age group. This is similar to a recent study of 10,222 16-year-old twins which estimated the heritability of sleep quality to be 42% [32].

Minutes of wake after sleep onset (WASO) and sleep efficiency (SE) can also be considered markers of sleep quality. Recent heritability estimates for WASO ranged from 73 to 85% [30, 33] and 55 to 80% for SE [29••, 30]. Inderkum and Tarokh [30] also found that estimates differed depending on whether sleep was measured on school days or free days, but the differences were not as marked as those found for sleep duration. Lower heritability estimates for WASO and SE were observed in a recent EEG study in adolescents, but this had a small sample size (*N* = 60) [33].

#### Circadian Parameters

In a study of 608 8-year-olds, Breitenstein et al. [29••] assessed circadian timing and variability of sleep using actigraphy. They did not find evidence that sleep midpoint and sleep midpoint variability were explained by genetic factors, and most of the variance was explained by shared environmental factors. Heritability estimates for chronotype in adolescents (11–14 years) were found to be higher on free days (87–91%) compared to school days (14%) when assessed using actigraphy [30], and 76% when assessed using a chronotype questionnaire. However, the small sample size (*N* = 51) means that there is unlikely to be power to obtain stable estimates. Gehrman and colleagues derived numerous circadian parameters from actigraphy data in 142 twins aged between 16 and 40 years [34•]. They estimated heritability using Sequential Oligogenic Linkage Analysis Routines. Circadian heritability estimates that remained significant after correcting for age and sex were primary minimum phase (88%), relative amplitude (57%), and M10 (10 h with maximal activity) (64%). However, there was a broad age range and estimates were not conducted in adolescents/young adult samples separately.

One recent study assessed the heritability of biological markers of circadian rhythms. Ouellet-Morin and colleagues [35] examined the cortisol awakening response (CAR), awakening level, and cortisol change from morning to evening (diurnal change) in 592 14-year-old twins. The genetic influence for CAR, awakening level, and diurnal change was estimated at 49.5% (A + D), 27.8% (A), and 31.4% (A), respectively. Using multivariate analysis, the authors found that the influence of A and D for CAR was 39.5% and 10%, respectively.

#### Sleep Architecture

The study by Gehrman et al. [34•] described above also derived duration of “light sleep” and “deep sleep” behavioral states from actigraphy using an algorithm based on second-by-second variability in movement. After correcting for age and sex, heritability for the duration of “light sleep” and “deep sleep” was 41% and 21%, respectively. Of note, the creators of the algorithm used to define light and deep sleep caution that these behavioral states do not correspond well with PSG-defined sleep stages [36]. Several high-density EEG studies have also been conducted in adolescents. Markovic et al. [37] measured sleep in 50 adolescents (mean age 13.2 years, range = 10–15). They assessed the distribution of EEG power (activity of different EEG frequencies) during NREM and REM sleep. In contrast to findings from adult studies [38], heritability estimates were low for delta (1–4.6 Hz) and sigma (11–16 Hz) bands in NREM (0.12 ≤ h^2^ ≤ 0.2) and REM sleep (0.01 ≤ h^2^ ≤ 0.2). There was higher heritability for beta bands (16.2–24 Hz) in NREM (0.48 ≤ h^2^ ≤ 0.51) and REM sleep (0.52 ≤ h^2^ ≤ 0.57). This emphasized the importance of examining high frequency ranges in EEG studies and the effect of shared environmental influences on other frequency bands. Markovic and colleagues [39] also examined the heritability of sleep EEG coherence in 62 adolescents (mean age = 12.5 years). EEG coherence measures connectivity based on correlation of EEG signals at a specific frequency and is a potential indicator of increased myelination and rewiring of the brain during development [40]. Across frequencies and sleep states, the heritability of sleep EEG coherence was 19%, with stronger contributions for unique environmental factors (median value range: 45% ≤ E ≤ 75%). The authors found that EEG coherence was strongest for sleep spindles which showed on average a heritability of 48% across connections. Finally, Rusterholz and colleagues [33] used high-density EEG to estimate the heritability of several sleep parameters in 60 adolescent twins (mean age = 12.46 years). Most sleep parameters did not show strong genetic influences: number of minutes of REM sleep, sleep efficiency, and total sleep time showed high unique environmental influences (81% ≤ E ≤ 98%), and variance in minutes of stage 1 sleep was primarily influenced by shared environmental factors (*C* = 85%). Moderate heritability was observed for duration of slow-wave sleep (37%), REM sleep latency (40%), and sleep efficiency (32%). When assessing EEG power in posterior regions of the brain, the authors found that 80–90% of the variance in slow oscillations, slow wave, and spindle activity was due to genetic factors. However, EEG power in anterior regions was primarily driven by shared environmental factors.

#### Insomnia

In 10,022 16-year-old twins, Madrid-Valero et al. estimated the heritability of insomnia symptoms (assessed using the Insomnia Severity Index) to be 41% [32]. Shakoor et al. [41] repeated these analyses in a smaller sample from the same study (*N* = 7,442) and found that the heritability for insomnia was lower for boys (34%) than girls (42%). Madrid-Valero et al. [42] also found that the heritability for Child Behaviour Checklist (CBCL) items “sleeps less than most kids” and “trouble sleeping” was 85% and 62%, respectively, in 2,060 children (age 8.06 years, range = 6–12). Other twin studies have examined the heritability of sleep onset latency (SOL), which in cases might be relevant to sleep onset insomnia. Recent studies using actigraphy estimate SOL heritability at 30% at age 8 [29••] and 48–77% at age 12 [30]. This is within the same range as a recent EEG study in 12–14-year-old adolescents, which estimated SOL heritability at 72% (although the *N* in this study was small, *N* = 60) [33].

#### Other Sleep-Related Difficulties and Disorders

Breitenstein et al. [29••] estimated the heritability of daytime sleepiness to be 27% in 608 children (age 8 years, *SD* = 0.63) using parent reports. In addition, Champion et al. [43] assessed pediatric restless legs syndrome (RLS) assessed by parent questionnaire and rated against 4 essential criteria defined by the International RLS Study Group in 2,033 twins aged 10.5 years (range 3–18). They found that heritability differed according to RLS subtype, being 14% for painless RLS but 64% for painful RLS. Breitenstein et al. [44] assessed sleep problems at age 5 in 406 twins (age 4.8 years, *SD* = 0.39) using total scores on the Child Sleep Habits Questionnaire [45]. Heritability for the total score was estimated at 28%. As outlined above, Madrid-Valero et al. [42] assessed sleep problems using individual items from the CBCL in 2,060 children (age 8.06 years, range = 6–12). In addition to the aforementioned insomnia-related items, heritability estimates for the other CBCL items were as follows: “sleeps more than most kids” (89% [ADE model]), “sleeps less than most kids” (85%) and “overtired” (83%), “nightmares” (73%), “talks or walks in sleep” (72%), and “trouble sleeping” (62%).

### GWAS

Marinelli et al. [46] conducted a GWAS of sleep duration in 10,554 children aged 2–14 years from the EAGLE consortium. SNP heritability of sleep duration was estimated at 14% after adjusting for age, sex, and principal components. In 2019, Dashti et al. [47] compared the results of this study with an adult sleep duration GWAS (SNP heritability 9.8%). They found that none of the 78 genome-wide significant loci identified in the adult GWAS was replicated in the EAGLE GWAS, but 45 of the 77 loci discovered in the EAGLE study showed consistent directionality in the adult GWAS.

Ohi et al. [48••] conducted a GWAS of sleep problems using the total score from the Sleep Disturbance Scale for Children [49] in 9,683 children aged 9–10 years. SNP heritability was 11% in children of European ancestry and 14% in children of trans-ancestry [48••]. A factor analysis on scores from the SDSC was used to derive subscales representing common sleep disorders. The subscales and corresponding SNP heritability estimates for each subscale are summarized in Table 2, ranging from 2.1 to 22.9%. The GWAS for sleep breathing disorders (sleep apnea and snoring) and sleep hyperhidrosis (falling asleep sweating and night sweating) subscales could not significantly explain variances in these phenotypes.

**Table 2:** Summary of genome-wide association studies published in the last 5 years which focus on sleep phenotypes and include children/adolescents

Author	Year	Country	Sleep phenotype	Sample	*N*	Age, years (range)	% Female	h^2^_SNP_
Jorgensen et al. [50••]	2021	Denmark	Lifetime nocturnal enuresis defined according to ICD-10 criteria and redeemed desmopressin prescriptions	iPSYCH2012 Danish population-based cohort	3,882 cases 31,073 controls	Cases^a^: 19.96 (*IQR* = 16.71–22.87) Controls: 20.38 (*IQR* = 17.13–23.21)	Cases: 28 Controls: not reported	0.239 to 0.304 (assuming a nocturnal enuresis prevalence of 7–15%)
Marinelli et al. [46]	2016	Finland, Spain, The Netherlands, UK	Parent rated question on sleep duration	EArly Genetics and Life course Epidemiology (EAGLE) Consortium	10,554 discovery 1,250 replication	10.56 (range = 2–13)	Not reported	0.14
Ohi et al. [48••]	2021	USA	SDSC total score	Adolescent Brain Cognitive Development (ABCD) study	9683 (4920 European ancestry)	9.9 (range = 9–10)	47.7	.109 Eur .139 Trans-anc
Ohi et al. [48••]	2021	USA	SDSC subscale: Disorders of arousal or nightmares (sleepwalking, sleep terrors, and nightmares)	Adolescent Brain Cognitive Development (ABCD) study	9683 (4920 European ancestry)	9.9 (range = 9–10)	47.7	.021 Eur .131 Trans-anc
Ohi et al. [48••]	2021	USA	SDSC subscale: Disorders of initiating and maintaining sleep (sleep duration and latency, problems in falling asleep, and night awakenings)	Adolescent Brain Cognitive Development (ABCD) study	9683 (4920 European ancestry)	9.9 (range = 9–10)	47.7	.119 Eur .139 Trans-anc
Ohi et al. [48••]	2021	USA	SDSC subscale: Disorders of excessive somnolence (daytime somnolence and restless sleep)	Adolescent Brain Cognitive Development (ABCD) study	9683 (4920 European ancestry)	9.9 (range = 9–10)	47.7	.170 Eur .229 Trans-anc
Ohi et al. [48••]	2021	USA	SDSC subscale: Sleep breathing disorders (sleep apnea and snoring)	Adolescent Brain Cognitive Development (ABCD) study	9683 (4920 European ancestry)	9.9 (range = 9–10)	47.7	.000 Eur .000 Trans-anc
Ohi et al. [48••]	2021	USA	SDSC subscale: Sleep hyperhidrosis (falling asleep sweating and night sweating)	Adolescent Brain Cognitive Development (ABCD) study	9683 (4920 European ancestry)	9.9 (range = 9–10)	47.7	.000 Eur .000 Trans-anc
Ohi et al. [48••]	2021	USA	SDSC subscale: Sleep–wake transition disorders (hypnic jerks, rhythmic movement disorders, and hypnagogic hallucinations)	Adolescent Brain Cognitive Development (ABCD) study	9683 (4920 European ancestry)	9.9 (range = 9–10)	47.7	.028 Eur .055 Trans-anc

Note. *ICD-10*, International Classification of Diseases (10th revision); *IQR*, interquartile range; *h*^*2*^_*SNP*_, single nucleotide polymorphism-based heritability estimate; *SDSC*, Sleep Disturbance Scale for Children

^a^Median age at first diagnosis = 8.12 (*IQR* = 6.71–10.10), median age at first redeemed prescription = 7.29 (*IQR* = 6.35–8.70), *Eur*, European; *Trans-anc*, trans-ancestry

One other GWAS was conducted by Jørgensen et al. [50••], who assessed lifetime nocturnal enuresis diagnosis in a Danish population using ICD-10 criteria and redeemed desmopressin prescriptions (3,882 cases, 31,073 controls). SNP heritability ranged from 23.9 to 30.4% assuming a nocturnal enuresis prevalence of 7–15%. This is also the first GWAS to identify genome-wide significant loci for nocturnal enuresis and to identify a genetic overlap with attention-deficit hyperactivity disorder.

## Future Directions

The studies in this review highlight several key areas for further consideration. First, most research utilized twin studies. Only 3 sleep GWAS in young people were published in the last 5 years and these had much smaller sample sizes compared to adult GWAS. There is evidence that heritability of sleep traits varies by age group [28••, 30, 51, 52]; therefore, future GWAS should examine genetic influences across different developmental stages, ideally using longitudinal data. Mendelian randomization (MR) studies often use SNPs identified in GWAS to infer causal relationships (e.g., between insomnia and depression). This assumes SNPs index sleep phenotypes occurring *before* onset of the disorder in question. However, most MR studies use SNPs from UK Biobank GWAS, with participants aged 40–69 years [53]. Existing comparisons between child and adult sleep GWAS suggest different SNPs are associated with sleep traits in childhood and adulthood [47]. It is therefore questionable whether MR studies using SNPs associated with midlife sleep phenotypes can adequately assess causal relationships between sleep and disorders which commonly develop in childhood or adolescence (e.g., depression [54]). This also applies when examining associations between polygenic scores (PGS) for sleep traits and other phenotypes in childhood and adolescence, as PGS are often derived using adult GWAS summary statistics. Furthermore, GWAS often rely on questionnaire measures to assess sleep phenotypes, and the studies reviewed in this paper demonstrate how estimates of genetic influence vary considerably according to measurement. Large sleep GWAS in child and adolescent samples using a range of measurement methods are therefore vital.

Second, the reviewed studies highlighted several factors to consider in future research. These include ancestral group [48••], whether sleep is measured on weekdays or free days [30], and method or reporter used to measure sleep [28••, 29••]. However, differences according to measurement method could be driven by unstable estimates, as twin studies using objective measures of sleep often used small sample sizes (*N* < 150) and are likely to be underpowered. Larger studies using objective sleep measures are needed. We also found that only two studies assessed sleep disorders using diagnostic criteria [43, 50••]. Most studies focused on traits related to insomnia, and future research examining other disorders in childhood and adolescence is needed. In addition, few studies reported heritability estimates by gender despite evidence that there are gender differences in sleep EEG characteristics [55] and risk for insomnia [56]. Recent meta-analyses of twin studies have not found evidence that heritability for sleep duration, sleep quality, or insomnia is moderated by sex [28••, 51], but this could be due to many studies not reporting results stratified by gender [51].

### Heritability Estimates

A recent study found that heritability estimates are often misunderstood, even amongst those with genetics training [22]. As our understanding of the role of genetics on sleep progresses, it is increasingly important for researchers to effectively communicate these findings to the public. For example, heritability estimates do not assess the influence of de novo mutations, as by definition they are not inherited, and most studies estimate narrow sense heritability [57] which assumes the absence of gene–gene and gene-environment interactions. Furthermore, SNP heritability does not consider the impact of rare genetic variants, which may have a greater impact than common variants [58]. These factors and other mechanisms such as epigenetic inheritance [59] may explain why SNP heritability estimates are often much lower than those from twin studies. Some researchers have questioned whether it is possible to separate genetic from environmental influences on complex traits due to the complex way in which genes and environment interact over time and have argued that there should be less focus on refining heritability estimates and more on understanding this complexity [57].

### Environmental Influences and Other Mechanisms

The reviewed studies demonstrate that environmental influences often play a large role in explaining variance in sleep phenotypes. Adoption studies and other genetically sensitive designs can disentangle the effects of environment from inherited factors. A key example is a study by Lewis and colleagues, who examined familial transition of depression by analyzing data from children conceived through in vitro fertilization who were either genetically related or unrelated to their parents [60]. They found that the correlations between parent and child depressive symptoms were similar regardless of biological relatedness, suggesting that environmental influences play a role in the intergenerational transmission of depression symptoms. We are not aware of any studies that have used this approach to examine intergenerational transmission of sleep phenotypes, but this provides a fruitful avenue for future work.

Identifying these environmental influences across different ages and sleep phenotypes will be crucial in understanding the aetiology of sleep disorders. There are multiple candidate environmental influences on child and adolescent sleep, including the use of digital media, diet/caffeine intake, parent-imposed bedtimes and sleep habits, and psychosocial changes during adolescence [2, 16, 61, 62]. Future research could examine genetic influences on these “environmental traits.” Recent studies have implicated other mechanisms such as bullying [41], sibling conflict [44], internalizing problems [32], antisocial behavior [42], emotional regulation [63], and impulsivity and anger [64]. There have also been studies in young adults that have examined pre-sleep arousal [65], dysfunctional beliefs about sleep [66], and loneliness [67] as other pathways that affect sleep. Future work could explore how these factors influence sleep in younger populations.

Environmental factors can also change heritability estimates over time, even for traits with a high genetic influence [68], and this should be considered in future research. Key factors to consider are increases in the use of digital technology, lifestyle changes due to the COVID-19 pandemic (e.g., more indoor activities and reduced sunlight exposure) [69], and long-term sleep disturbances arising from contracting COVID-19 [70]. Understanding gene-environment interactions and epigenetic influences will also be important. For example, insomnia is thought to develop because individuals with a predisposition to insomnia are more likely to develop insomnia after precipitating factors (e.g., stress) [71]. Palagini and colleagues hypothesized that epigenetic mechanisms may predispose individuals to insomnia, either through early life experiences or transgenerational epigenetic inheritance, and may also be involved in the maintenance of insomnia [72]. Epigenetic influences have also been implicated in other sleep disorders [73]. A recent study in 10-year-old children found that sleep duration measured using actigraphy was associated with DNA methylation patterns in a region of the genome implicating genes previously identified in sleep GWAS [74•]. Understanding how epigenetic mechanisms influence sleep in childhood and adolescence could be a promising avenue to inform our understanding of sleep and sleep disorders.

## Conclusion

Our review of recent literature highlights that more research is needed to understand how genetic factors influence sleep and sleep disorders across different ages in childhood and adolescence. This is particularly the case for GWAS, which are primarily conducted in adults. Further understanding of how genes interact with the environment and other psychological and biological mechanisms will be crucial to inform our understanding of the aetiology of sleep disorders and inform interventions to improve sleep in young people.
